# GPs’ views on green social prescribing in Scotland: analysis of a national cross-sectional survey

**DOI:** 10.3399/BJGPO.2024.0259

**Published:** 2025-09-24

**Authors:** Helen Frost, Tricia R Tooman, Bruce Mason, Eddie Donaghy, Katie Hawkins, Sue Lewis, Maria Wolters, Stewart W Mercer

**Affiliations:** 1 Advanced Care Research Centre, Usher Institute, University of Edinburgh, Edinburgh, UK; 2 Usher Institute, University of Edinburgh, Edinburgh, UK; 3 The Access Place, NHS Lothian, Edinburgh, UK; 4 School of Informatics, University of Edinburgh, Edinburgh, UK; and OFFIS Institute for Information Technology, Oldenburg, Germany

**Keywords:** general practice, green social prescribing, social prescribing, multimorbidity

## Abstract

**Background:**

Green social prescribing (GSP) aims to link patients to nature-based health interventions (NBHIs) through GPs. However, knowledge of GPs’ views on GSP is limited.

**Aim:**

To explore GPs’ views on GSP and the factors influencing these views.

**Design & setting:**

National cross-sectional survey of GPs’ working lives in Scotland, conducted in 2023, which included four questions about GSP.

**Method:**

Descriptive analysis of GPs’ views of GSP and univariate and multivariate (binary logistic) analysis of factors influencing these views.

**Results:**

The survey found 79.6% (*n* = 1098) of GPs had heard of GSP, 81.3% (*n* = 1106) would be happy to refer patients to NBHIs, 67.8% (*n* = 931) thought GSP was suitable for older patients with multimorbidity, and 43.7% (*n* = 599) felt that patients living in deprived areas would access GSP. Greater knowledge of GSP was associated with White ethnicity (adjusted odds ratio [aOR] 2.04; 95% confidence interval [CI] = 1.30 to 3.22, *P* = 0.002) and the number of clinical sessions worked per week (aOR 0.90; 95% CI = 0.82 to 0.99, *P* = 0.034). Higher job satisfaction was associated with more positive views about the suitability of GSP for older patients with multimorbidity (aOR 1.14; 95% CI = 1.00 to 1.30; *P* = 0.043) as were views on whether patients living in deprived areas would access GSP (aOR 1.20; 95% CI = 1.03 to 1.33, *P* = 0.013). GPs working in deprived areas also had more positive views regarding whether patients living in deprived areas would access GSP (aOR 1.24; 95% CI = 1.06 to 1.45, *P* = 0.159).

**Conclusion:**

GPs in Scotland are aware of and willing to refer to GSP but have concerns about accessibility for patients from deprived areas. Views were influenced by personal and practice characteristics.

## How this fits in

Green social prescribing (GSP) is a form of social prescribing that aims to link patients to nature-based health interventions (NBHI) via GPs. However, little is known about GPs’ knowledge and attitudes towards GSP and whether they view it as suitable for patients with complex needs, such as older patients with multimorbidity or those living in deprived areas. We found that most GPs were aware of GSP and would be happy to refer patients; more than two-thirds of GPs felt that GSP would be suitable for older patients with multimorbidity, but fewer than half thought that patients from deprived areas would access GSP. Views were influenced by ethnicity, sessions worked per week, job satisfaction, and whether the practice was in an area of high deprivation.

## Introduction

Owing to the ageing population in Scotland and in most countries around the world, multimorbidity is one of the ‘grand challenges’ facing health and social care.^1^ Similarly, health inequalities are a major problem in Scotland, which has the widest health inequalities in Western Europe, with the inequality gap widening year after year. Thus innovative ‘new models of care’, such as green social prescribing (GSP), are urgently needed, and exclusion of vulnerable groups will widen inequalities further.

GSP is a form of social prescribing that aims to link patients to nature-based health interventions (NBHIs) via GPs. The benefits of NBHIs, such as accessing forests, parks, community gardens, and beaches, are increasingly apparent^2–5^ and include improved physical and mental health in different populations, such as people with anxiety, depression, cardiometabolic health problems, and poor cognition.^2,6–8^ Most importantly, NBHIs are likely to benefit people living in lower socioeconomic groups more than affluent people.^9^

GSP may also be a way of reducing health inequalities, but patients living in deprived areas often face barriers to accessing NBHIs.^9,10^ Given these potential benefits, ‘green health’ policies have developed with an aim to make NBHIs more widely available across the UK at both a local and national level.^11–13^ The COVID-19 pandemic heightened awareness that being outdoors in nature benefited mental health and consequently GSP is gaining support across the UK. In England a £5.77 million cross-government funded project was rolled out in 2020 to focus on developing NBHIs to improve mental health and wellbeing.^14^ An evaluation of this project demonstrated statistically significant improvements in wellbeing and a positive social return on investment.^14^ In Scotland ‘Our Natural Health Service’ policy aimed to support services across a range of nature-based activities in collaboration with national organisations, to bring health and social services, environment, and third sector organisations closer together.^11^ However, referral pathways, funding, and availability of NBHIs vary across the four nations of the UK^10,11,13,15,16^ and a lack of awareness and knowledge of GSP is cited as a key barrier to progressing GSP in primary care.^5,14^

A few studies have examined GPs’ characteristics, such as sex, in relation to those who advocate for GSP.^12,17^ Female clinicians tend to be more in favour of referring patients to nature-based activity and they are also more likely to refer female patients.^12,17^ In addition, clinicians who spend more time outdoors and in nature themselves tend to be more in favour of referral to GSP than those who spend less time outdoors.^12^ However, these studies do not account for other individual and practice factors that may influence such views. There is limited knowledge about whether GPs view GSP as worthwhile for patients with complex needs, such as older patients with multimorbidity or those living in deprived areas.

The aim of this study was to explore the following questions:

1. What are GPs’ knowledge and views on referring patients to GSP in Scotland?
2. What are their views on the suitability of GSP for older patients with multimorbidity?
3. What are their views on the accessibility of GSP for patients living in deprived areas?
4. How do these views vary by GPs’ individual and practice characteristics?

## Method

A national survey questionnaire of GPs’ working lives and views was posted to all qualified GPs in Scotland in October 2023, with two postal follow-ups between December 2023 and January 2024.^18^ The focus of the survey was to assess GPs’ working lives, future work intentions, and views on the Scottish GP contract. Four questions on GPs’ knowledge and attitudes towards GSP were included in the survey as follows:

*‘Green prescribing is a type of social prescribing that aims to help patients access green spaces and activities such as gardening, nature, walks, etc.*

*Q1. Is this something you have heard of?* (Answered ‘yes’ or ‘no’).

*Q2. Would you refer patients to such activities?* (Answered ‘yes’ or ‘no’).

*Q3.Do you think green prescribing would be suitable for older patients with multimorbidity?* (Answered ‘yes’, ‘no’, or ‘don’t know’).

*Q4. Do you think patients living in deprived areas would access green prescribing?* (Answered ‘yes’, ‘no’, or ‘don’t know’).’

Information was collected on GP demographics and employment details. GPs’ individual variables included age, sex, ethnicity, if they were a partner in the practice, years in their current practice, clinical sessions worked per week, and job satisfaction. Practice variables included practice list size (number of registered patients), and the deprivation level of patients registered by the practice. Deprivation was measured by the Scottish Index of Multiple Deprivation (SIMD).^19^ Rurality was based on the proportion of registered patients per practice living in urban or rural areas.^20^ In total, *n* = 1385/4529 GPs (31%) responded to the survey.

### Analysis

For the analysis, Q3 and Q4 were coded as ‘yes’ and ‘no or don’t know’ for ease of interpretation. First, univariate analysis of the associations between knowledge and attitudes to GSP and the GPs’ individual and practice characteristics was conducted using SPSS (version 27). We then conducted multivariate analysis by binary logistic regression, with the dependent variables being knowledge and views on GSP (with Q1 and Q2 dichotomised as ‘yes’ = 1 and ‘no’ = 0, and Q3 and Q4 dichotomised as ‘yes’ = 1 and ‘no/don’t know’ = 0) and with the GP’s individual (age, gender, ethnicity, partner in practice or not, years in current practice, clinical sessions worked per week, and job satisfaction) and practice characteristics (list size, SIMD score, rural or urban locality) entered as independent predictors. No imputation of missing data was carried out on the data.

## Results

The representativeness of responders to all qualified GPs in Scotland based on available characteristics are shown in Table 1. Responders (*n* = 1376/1385; missing data = 9) included 586 males (42.6%,) 788 females (57.3%), and two who identified as other (0.1%); 1254 out of 1371 identified as White (91.5%), 117 (8.5%) identified as ethnic minority; the mean age was 47 years (range 29 years–80 years). The average number of sessions worked was 6.5 (range 1–13), and the average length of time in current practice was 12 years (range 6 months–43 years). Further details of responders’ characteristics can be found in Tables 1 and 2. Overall, four out of five GPs (*n* = 1098 out of 1378, 79.6%) had heard of GSP, and a similar number (*n* = 1106 out of 1360, 81.3%) would refer patients to NBHI (Table 2). More than two-thirds (*n* = 931 out of 1373, 67.8%) thought GSP was suitable for older patients with multimorbidity, whereas only 43.7% (*n* = 599 out of 1370) felt that patients living in deprived areas would be able to access GSP (Table 2).

**Table 1.** Representativeness of 2023 GP survey compared with all GPs in Scotland

	Scotland (2022) *n* = 4515	GP survey (2023) *n* = 1378
Age, years		
<35	337 (7%)	108 (8%)
35–44	1864 (41%)	403 (29%)
45–54	1454 (32%)	510 (37%)
55–64	789 (17%)	260 (19%)
≥65	66 (1%)	13 (1%)
Female gender	3217 (71%)	788 (57%)
GP partner	3236 (72%)	1143 (83%)
High deprivation	903 (20%)	276 (20%)
Urban locality	3296 (73%)	957 (69%)

*n* (%) excludes missing data. Deprivation is calculated as the proportion of patients in the most deprived quintile of all practices in Scotland, calculated from the percentage of registered patients who are living in the top 15% most deprived areas based on the Scottish Index of Multiple Deprivation (SIMD). Urban locality is based on the proportion of registered patients living in urban conurbations as defined by the Scottish Government. Data on all GPs in Scotland in 2022 is available at; https://publichealthscotland.scot/publications/general-practice-gp-workforce-and-practice-list-sizes/general-practice-gp-workforce-and-practice-list-sizes-2012-2022/#:~:text=After%202018%2C%20the%20number%20of,high%20of%2034%25%20in%202013 .

**Table 2.** Univariate analysis of the associations between knowledge and attitudes to green social prescribing (GSP) and the GPs’ individual and practice characteristics

	Q1: Heard of green prescribing		Q2: Would refer patients		Q3: Suitable for older patients with multimorbidity		Q4: Patients in deprived areas would access	
	Yes	*P* value	Yes	*P* value	Yes	*P* value	Yes	*P* value
All GPs	1098 (79.6%)	-	1106 (81.3%)	-	931 (67.8%)	-	599 (43.7%)	
Age group:								
<35 years	85 (79.4%)	0.748	92 (86.0%)	0.125	78 (72.9%)	0.386	45 (42.1%)	0.146
35–44 years	330 (81.9%)		336 (84.0%)		281 (69.7%)		188 (46.9%)	
45–54 years	406 (79.6%)		399 (79.2%)		333 (65.8%)		213 (42.2%)	
≥55 years	215 (79.8%)		209 (79.2%)		186 (68.4%)		112 (41.2%)	
Gender:								
Female	640 (81.3%)	0.068	651 (84.0%)	0.004	536 (68.5%)	0.829	336 (43.1%)	0.339
Male	435 (77.3%)		450 (77.9%)		392 (67.1%)		261 (44.7%)	
Ethnicity:								
White Caucasian	1012 (80.8%)	<0.001	1009 (81.6%)	0.350	846 (67.8%)	0.713	549 (44.1%)	0.645
Other	78 (66.7%)		89 (78.1%)		80 (69.6%)		40 (40.4%)	
Practice partner:								
Yes	907 (79.6%)	0.650	898 (80.0%)	0.130	761 (67.2%)	0.017	490 (43.3%)	0.097
No	189 (79.4%)		206 (87.3%)		169 (71.0%)		108 (45.6%)	
Years in practice:								
<10	522 (79.6%)	0.925	547 (84.2%)	0.010	448 (68.4%)	0.649	306 (46.9%)	0.011
≥10	572 (79.8%)		553 (78.7%)		481 (67.7%)		292 (41.1%)	
Sessions per week:								
<6	589 (81.2%)	0.105	599 (83.8%)	0.012	508 (70.6%)	0.106	325 (45.2%)	0.866
≥6	502 (77.7%)		499 (78.5%)		420 (65.2%)		273 (42.5%)	
List size:								
<5000	237 (77.7%)	0.333	251 (83.4%)	0.558	217 (71.6%)	0.212	147 (48.8%)	0.213
5000–10 000	572 (81.1%)		561 (80.5%)		474 (67.7%)		303 (43.3%)	
>10 000	289 (78.1%)		294 (81.2%)		240 (64.9%)		149 (40.4%)	
Deprivation:								
Low	366 (82.6)	0.140	344 (78.5%)	0.160	308 (70.0%)	0.343	178 (40.5%)	<0.001
Medium	373 (77.5%)		389 (81.9%)		328 (68.6%)		203 (42.6%)	
High	481 (78.7%)		373 (83.4%)		295 (64.8%)		218 (48.0%)	
Rurality:								
Urban	920 (79.4%)	0.804	931 (81.7%)	0.460	774 (67.2%)	0.301	502 (43.7%)	0.328
Rural	178 (80.2%)		175 (79.5%)		157 (71.0%)		97 (44.1%)	
Job satisfaction:								
Above average	566 (80.1%)	0.958	578 (83.2%)	0.009	496 (70.6%)	0.249	320 (45.6%)	0.527
Average or below	502 (79.1%)		502 (79.9%)		408 (64.5%)		260 (41.1%)	

*n* (%) excludes missing data.

Univariate analysis of the associations between knowledge and attitudes to GSP and the GPs’ individual and practice characteristics are also shown in Table 2. GPs of White ethnicity (White Caucasian, *n* = 1012 (80.8%), others, *n* = 78 (66.7%); *P*<0.001) were significantly more likely to have heard of GSP than GPs of other ethnic backgrounds (Q1). Female GPs (females, *n* = 651 [84.0%], male, *n* = 450 [77.9%]; *P* = 0.004), GPs with less than 10 years’ experience in their current practice (<10 years, *n* = 547 [84.2%],>10 years, *n* = 553 [78.7%]; *P* = 0.01), those working less than 6 sessions a week (<6 sessions, *n* = 599 [83.8%], ≥6 sessions, *n* = 499 [78.5%]; *P* = 0.012), and those with higher job satisfaction (above average, *n* = 578 [83.2%]; below average, *n* = 502 [79.9%]; *P* = 0.009) were more likely to refer patients to GSP (Q2). GPs who were not partners in the practice were more likely to feel that older patients with multimorbidity would be suitable for GSP (partners, *n* = 761 [67.2%], not partners, *n* = 169 [71.0%]; *P* = 0.017) (Q3). GPs working in deprived areas (low deprivation, *n* = 178 [40.5%], medium deprivation, *n* = 203 [42.6%], high deprivation, *n* = 218 [48.0%]; *P*<0.001) and those with less than 10 years in their current practice (<10 years, *n* = 306 [46.9%], >10 years, *n* = 292 [41.1%]; *P* = 0.011) were more likely to have a positive response regarding whether patients in deprived areas would access GSP (Q4).

Table 3 shows the variables that were independently predictive of positive answers to questions on GSP in the binary logistic regression. Ethnicity remained a significant predictor of knowledge of GSP (Q1), with GPs of White ethnicity being more than twice as likely to have heard of GSP than those of other ethnicities (adjusted odds ratio [aOR] 2.04, 95% confidence interval [CI] = 1.30 to 3.22; *P* = 0.002). In addition, GPs who worked more clinical sessions per week were less likely to have heard of GSP (aOR 0.90, 95% CI = 0.82 to 0.99; *P* = 0.034). No factors emerged as being significant with regard to whether GPs would refer patients to GSP (Q2). For Q3, GPs who worked more sessions a week were less positive (aOR 0.9, 95% CI = 0.83 to 0.98; *P* = 0. 019), and those with higher job satisfaction more positive (aOR 1.14, 95% CI = 1.00 to 1.30; *P* = 0.043) about the suitability of GSP for older patients with multimorbidity. For Q4, views on whether patients in deprived areas would access GSP were also more positive for GPs with higher job satisfaction (aOR 1.20, 95% CI = 1.03 to 1.33; *P* = 0.013) as were those working in deprived areas (aOR 1.24, 95% CI = 1.06 to 1.45; *P* = 0.008).

**Table 3.** Multivariate analysis of positive predictors of knowledge and attitudes to GSP

	Predictor	Adjusted odds ratio	Lower and upper 95% CIs	*P* value
				
Q1: Heard of green prescribing	White ethnicity	2.04	1.30 to 3.22	0.002
	Sessions per week	0.90	0.82 to 0.99	0.034
Q.3 Suitable for older patients with multimorbidity	Sessions per week	0.90	0.83 to 0.98	0.019
	Job satisfaction	1.14	1.00 to 1.30	0.043
Q4: Patients in deprived areas would access	Job satisfaction	1.20	1.03 to 1.33	0.013
	Deprivation	1.24	1.06 to 1.45	0.008

Independent variables entered into the logistic regressions were age, gender, ethnicity, partner in practice, years in current practice, sessions worked per week, practice list size, practice deprivation, practice rurality, and job satisfaction. In all regression models, the Hosmer–Lemeshow test was statistically insignificant (*P*<0.05).

## Discussion

### Summary

This study is the first to report on GPs’ views of GSP in a national survey. Most GPs were aware of GSP and would be happy to refer patients to NBHIs. More than two-thirds felt that GSP would be suitable for older patients with multimorbidity but fewer than half thought that patients from deprived areas would access NBHIs. After controlling for other individual and practice characteristics, GPs from ethnic minority backgrounds remained significantly less aware (*P*<0.002) , and GPs who worked fewer sessions per week significantly more aware of GSP (*P*<0.002) but were just as willing as other GPs to refer patients to NBHIs. In terms of the suitability of older patients with multimorbidity for GSP, GPs who worked more sessions a week were less positive and those with higher job satisfaction were more positive. GPs with higher job satisfaction and those who worked in deprived areas were also more positive about whether patients in deprived areas would access GSP.

### Strengths and limitations

A key strength of this survey was that it included all qualified GPs in Scotland, with a relatively good response rate for GP surveys, and responders were broadly nationally representative in terms of age, gender, whether a partner or not, practice deprivation, and rurality. The focus of the survey was on a wide range of workforce factors and views relevant to GPs’ working lives, including the new Scottish GP contract. Furthermore, given that it was not explicitly focused on GSP (four questions in the 10-page survey), those who responded are unlikely to be biased advocates for GSP.

Although the sample size was quite large (1378), some of the subgroups were limited in numbers. For instance, the results of our study on ethnicity should be viewed with some caution owing to the small sample size of the GPs from ethnic minorities. In addition, there were only four dichotomous questions on GSP and therefore deeper exploration of the rationale for their response was not possible. We do not know if the GPs had previously referred patients to GSP, only whether they believed they would. Including more detailed questions would have provided more information but we were limited in the number of questions we could include on GSP, as the main focus of the survey was about GP working life and job satisfaction.^18^ Future work is required to explore GPs’ views on GSP in more depth, and the use of Likert scales rather than dichotomous answers in any future work should be considered.

### Comparison with existing literature

Our overall findings are comparable with a study in England that found 76% of clinicians had heard of GSP.^12^ Regarding ethnicity, 72% of White clinicians compared with 61% of clinicians from ethnic minority backgrounds in England were more favourable towards GSP.^12^ This contrasts with our findings that GPs of ethnic minority backgrounds were as willing to refer patients as White GPs, but fewer had heard of GSP. This finding is notable particularly as other research shows that ethnic minority patient groups have more difficulties accessing primary care and have poorer health outcomes.^21^ If a GP’s ethnic background influences awareness of the benefits of being in nature, this could further increase health inequalities. However, this finding requires a deeper understanding of the cultural variation and historic experiences that may influence how some people from ethnic minority backgrounds value being in nature^22^ and consequently how this might influence a GP’s decision to refer patients to NBHIs. This is important as evidence suggests that people from lower socioeconomic status and minority groups are less likely to have the opportunity to enjoy nature, yet they are more likely to benefit from GSP than other more affluent groups.^9,22^ Further research may be needed to understand these findings to be able to develop strategies to reduce ethnic inequalities in health care.^23^

The English study found that female clinicians were more positive about GSP than male clinicians^12^ and a pilot study of GSP in primary care reported that most prescribers to NBHIs were women,^17^ but in both studies there was no adjustment for other factors. Our results indicate the importance of taking GPs’ individual and practice characteristics into account when examining such associations.

It is of interest that most GPs in our study felt that older people with multimorbidity would be suitable for GSP, given the increasing prevalence of multimorbidity owing to the ageing population. Encouraging such patients to participate in GSP could lead to a range of beneficial outcomes, including reducing the severity of frailty.^24^ However, these findings were again modified by the working patterns of the GPs, and by job satisfaction. A positive association between GP job satisfaction and enthusiasm for social prescribing has been reported previously owing to GPs feeling that social prescribing increased their job satisfaction and benefited patients’ health and wellbeing.^25^ GPs in Scotland and across Europe are reporting increasing workloads leading to potential burnout^18,26^ and it is likely that they have less time to think enthusiastically about innovative interventions. Thus, this may be a reason why some GPs with low job satisfaction are less likely to refer patients to GSP. Conversely, GPs with a higher job satisfaction are less likely to suffer burnout^26^ and providing preventive care, such as GSP, is positively related to higher job satisfaction.^26^

It is also noteworthy that most GPs in our study did not think that patients from deprived areas would participate in GSP, but this was again modified by job satisfaction with more positive views for those with higher job satisfaction. GPs who worked in deprived areas were more likely to be positive. We suggest this is because they may have a deeper knowledge and understanding of their patients and their patients’ needs and are therefore more inclined to be optimistic about GSP compared with GPs working in more affluent areas whose views were possibly more speculative. This is an important finding to build on given that people from deprived areas stand to gain the most from GSP.^9^

### Implications for research and practice

Despite the apparent enthusiasm for GSP in our study, engaging GPs in the GSP referral process is challenging^5,25^ and GPs need additional support to provide new or innovative interventions. Although awareness of GSP is developing,^5,27^ more research is required for a greater understanding of the practical barriers to referring patients to GSP, especially in areas of high deprivation, to enable interventions and policy solutions to prevent a widening inequality gap. These barriers may include, for example, awareness across cultural and ethnic groups; the training needs of staff; improving motivation; patient health literacy; NBHI access issues such as transport, costs, and disability; along with sustainability and funding of NBHIs that are predominantly provided by third sector organisations. Such understanding can then lead to primary care-based interventions that support GPs to engage in GSP, thus enabling patients — especially those living in deprived areas — to access NBHIs and thereby reap the many health benefits associated with such activities.

## Data

The data that the conclusions rely on are presented in the article.

## References

[1] Mercer SW, Guthrie B, Furler J (2012). Multimorbidity and the inverse care law in primary care. BMJ.

[2] Coventry PA, Brown JE, Pervin J (2021). Nature-based outdoor activities for mental and physical health: systematic review and meta-analysis. SSM Popul Health.

[3] Harrison H, Burns M, Darko N, Jones C (2023). Exploring the benefits of nature-based interventions in socio-economically deprived communities: a narrative review of the evidence to date. Perspect Public Health.

[4] Adewuyi FA, Knobel P, Gogna P, Dadvand P (2023). Health effects of green prescription: a systematic review of randomized controlled trials. Environ Res.

[5] de Bell S, Alejandre JC, Menzel C (2024). Nature-based social prescribing programmes: opportunities, challenges, and facilitators for implementation. Environ Int.

[6] Lamont R, Hinson C (2024). A narrative review of reviews of nature exposure and human health and well-being in the UK (NEER030).

[7] Twohig-Bennett C, Jones A (2018). The health benefits of the great outdoors: a systematic review and meta-analysis of greenspace exposure and health outcomes. Environ Res.

[8] Nguyen P-Y, Astell-Burt T, Rahimi-Ardabili H, Feng X (2023). Effect of nature prescriptions on cardiometabolic and mental health, and physical activity: a systematic review. Lancet Planet Health.

[9] Rigolon A, Browning M, McAnirlin O, Yoon HV (2021). Green space and health equity: a systematic review on the potential of green space to reduce health disparities. Int J Environ Res Public Health.

[10] Frost H, Tooman T, Hawkins K (2023). Green social prescribing: challenges and opportunities to implementation in deprived areas. Br J Gen Pract.

[11] NatureScot & NHS Scotland (2020). Scotland’s outdoors. Our natural health service. https://www.nature.scot/sites/default/files/2020-04/Our%20Natural%20Health%20Service%20leaflet%20-%20April%202020.pdf.

[12] Department of Health and Social Care (2023). Exploring perceptions of green social prescribing among clinicians and the public.

[13] NHS England (2023). Green social prescribing.

[14] Haywood A, Dayson C, Garside R (2024). National evaluation of the Preventing and Tackling Mental Ill Health through Green Social Prescribing Project: final report — March 2021 to June 2023.

[15] Green Nature and Wellbeing (2023). Green and social prescribing.

[16] Northern Ireland environment link (2023). Social Prescribing Toolkit.

[17] Bradley E (2022). Nature prescriptions: supporting the health of people and nature. A report on the outcomes of an urban pilot of Nature Prescriptions in Edinburgh. RSPB. https://www.rspb.org.uk/about-us/annual-report/nature-boosts-health-and-wellbeing.

[18] Donaghy E, Sweeney KD, Ng L (2025). Primary care transformation in Scotland: a comparison of two cross-sectional national surveys of GPs’ views in 2018 and 2023. Br J Gen Pract.

[19] Scottish Government (2020). Scottish Index of Multiple Deprivation.

[20] Scottish Government (2020). Shaping the future together: Remote and Rural General Practice Working Group report.

[21] Hayanga B, Stafford M, Bécares L (2024). Ethnic inequalities in primary care experiences for people with multiple long-term conditions: evidence from the general practice patient survey. Public Health (Fairfax).

[22] Rishbeth C, Neal S, French M, Snaith B (2022). Included outside: engaging people from ethnic minority backgrounds in nature. Evidence Briefing: Natural England Technical Information Note TIN185. https://publications.naturalengland.org.uk/publication/4729021765255168.

[23] Hayanga B, Stafford M, Bécares L (2021). Ethnic inequalities in healthcare use and care quality among people with multiple long-term health conditions living in the United Kingdom: a systematic review and narrative synthesis. Int J Environ Res Public Health.

[24] Elston J, Gradinger F, Asthana S (2019). Does a social prescribing “holistic” link-worker for older people with complex, multimorbidity improve well-being and frailty and reduce health and social care use and costs? A 12-month before-and-after evaluation. Prim Health Care Res Dev.

[25] Evers S, Kenkre J, Kloppe T (2024). Survey of general practitioners’ awareness, practice and perception of social prescribing across Europe. Eur J Gen Pract.

[26] Stobbe EJ, Groenewegen PP, Schäfer W (2021). Job satisfaction of general practitioners: a cross-sectional survey in 34 countries. Hum Resour Health.

[27] Fullama J, Hunt H, Lovell R (2021). A handbook for nature on prescription to promote mental health. https://www.ecehh.org/wp/wp-content/uploads/2021/05/A-Handbook-for-Nature-on-Prescription-to-Promote-Mental-Health_FINAL.pdf.

